# Motor Imagery during Action Observation of Locomotor Tasks Improves Rehabilitation Outcome in Older Adults after Total Hip Arthroplasty

**DOI:** 10.1155/2018/5651391

**Published:** 2018-03-19

**Authors:** Uros Marusic, Sidney Grosprêtre, Armin Paravlic, Simon Kovač, Rado Pišot, Wolfgang Taube

**Affiliations:** ^1^Institute for Kinesiology Research, Science and Research Centre Koper, Koper, Slovenia; ^2^Department of Health Sciences, Alma Mater Europaea-ECM, Maribor, Slovenia; ^3^EA4660, C3S Culture Sport Health Society, Université de Franche-Comté, Besançon, France; ^4^Valdoltra Orthopaedic Hospital, Ankaran, Slovenia; ^5^Department of Medicine, Movement and Sport Sciences, University of Fribourg, Fribourg, Switzerland

## Abstract

This study aimed at determining whether the combination of action observation and motor imagery (AO + MI) of locomotor tasks could positively affect rehabilitation outcome after hip replacement surgery. Of initially 405 screened participants, 21 were randomly split into intervention group (*N* = 10; mean age = 64 y; AO + MI of locomotor tasks: 30 min/day in the hospital, then 3×/week in their homes for two months) and control group (*N* = 11, mean age = 63 y, active controls). The functional outcomes (Timed Up and Go, TUG; Four Step Square Test, FSST; and single- and dual-task gait and postural control) were measured before (PRE) and 2 months after surgery (POST). Significant interactions indicated better rehabilitation outcome for the intervention group as compared to the control group: at POST, the intervention group revealed faster TUG (*p* = 0.042), FSST (*p* = 0.004), and dual-task fast-paced gait speed (*p* = 0.022), reduced swing-time variability (*p* = 0.005), and enhanced cognitive performance during dual tasks while walking or balancing (*p* < 0.05). In contrast, no changes were observed for body sway parameters (*p* ≥ 0.229). These results demonstrate that AO + MI is efficient to improve motor-cognitive performance after hip surgery. Moreover, only parameters associated with locomotor activities improved whereas balance skills that were not part of the AO + MI intervention were not affected, demonstrating the specificity of training intervention. Overall, utilizing AO + MI during rehabilitation is advised, especially when physical practice is limited.

## 1. Introduction

Prolonged immobilization and inactivity after injury and/or surgery may lead to serious motor and cognitive dysfunctions, especially in older adults [1, 2]. More precisely, immobilization in the acute period after hip arthroplasty has become more and more frequent in this population due to the increasing amount of people suffering from osteoporosis, which can result in hip fractures [3]. Despite the knowledge that immobilization not only negatively affects cardiovascular and pulmonary parameters but also increases the risk of falls and movement disorders [1, 4], patients are generally inactive during this period due to their limited ability to participate in physical exercise. In addition, immobilization leads to several impairments of motor function [5]. Motor impairments that follow short periods of inactivity are believed to be principally driven by changes occurring at the cortical level rather than the muscular level [6–8]. Indeed, a significant reduction of the cortical motor area representing the immobilized limb could be observed [9].

To counteract at least some of these risk factors, mental simulation techniques such as action observation (AO) or motor imagery (MI) have been proposed as feasible alternatives to stimulate the motor system [10, 11]. The AO therapy requires subjects to observe a video clip or watch actions performed by an operator [12, 13] while MI represents the mental simulation of motor actions without any corresponding motor output [14].

In the last decade, a growing number of AO- or MI-based interventions were successfully conducted that aimed at fostering rehabilitation of patients, for instance, after stroke [12, 15, 16], Parkinson's disease (for review, see [17]), or orthopedic injury and/or surgery [18, 19]. The use of AO in healthy participants has been shown to limit the reduction of brain area that is normally induced by immobilization [20]. Thus, activation of cerebral visuomotor systems during AO seems sufficient to counteract negative cortical plasticity induced by immobilization. Similarly, MI was demonstrated to counteract the slowdown of sensorimotor processes induced by short-term immobilization [21]. The reason for the efficiency of those mental simulation techniques is considered to rely on activation of overlapping brain areas during AO, MI, and physical execution of the motor task [14, 22]. Traditionally, AO and MI have been considered as independent intervention methods. Recently, however, more and more evidence emerged that proposes increased efficiency when combining AO with MI (AO + MI), meaning that MI is performed during AO (for reviews, see [23, 24]). In this context, AO + MI has revealed higher corticospinal excitability [25, 26] as well as greater activity in motor areas of the brain [27] compared to either AO or MI alone. Noteworthy some studies even reported oversummative activity [27, 28] compared to the sum of brain activity during independent AO and independent MI. These findings were interpreted as evidence that neural correlates of MI and AO might merge rather than compete with each other [24]. However, due to a lack of intervention studies, it is not clear to date whether the combination of AO and MI (AO + MI) in addition to common physical therapy has important implications for neurorehabilitation and motor (re-)learning in patients (for reviews, see [23, 24]).

The aim of the present study therefore was to evaluate AO + MI in a clinical setting by comparing the rehabilitation outcomes of patients undergoing an AO + MI intervention with those of patients that were treated in the conventional way only. One major aim of our study was to minimize the period of inactivity and to start with an early rehabilitation. Finally, our main hypothesis was that the combination of AO and MI (AO + MI) would result in better rehabilitation outcome than the conventional intervention alone.

## 2. Methods

### 2.1. Participants

**Figure 1:**
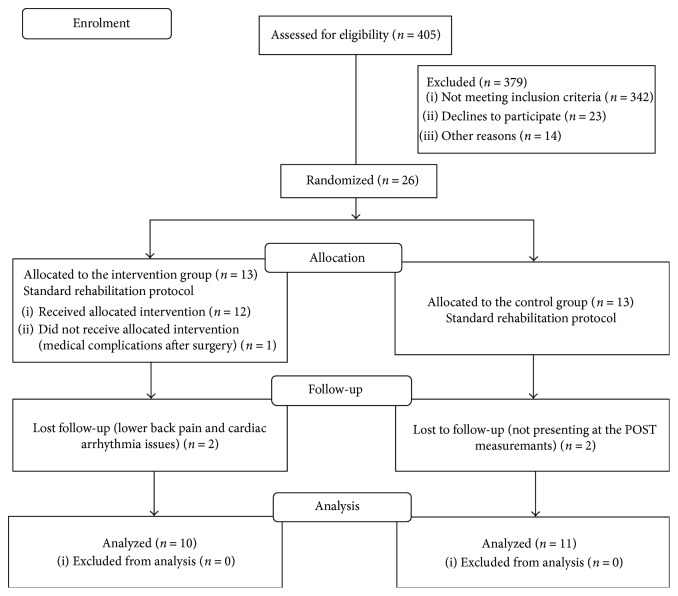
CONSORT flow diagram of the *PANGeA hip study, Valdoltra 2015*.

Participants were recruited from the general database of Valdoltra Orthopaedic Hospital, University of Primorska, Ankaran, Slovenia. From 405 subjects that were assessed for eligibility, 26 volunteered to participate in the “*PANGeA hip study, Valdoltra 2015*.” Finally, 21 participants successfully completed both (PRE and POST) measurements (for baseline characteristics, see Table 1). Due to primary osteoarthritis of the hip, the cementless total hip arthroplasty (THA) was performed through direct lateral approach in all participants. The enrolment, randomization, and final analysis procedures are shown in the CONSORT flow diagram (Figure 1). Prior to the study, all participants were physically screened by medical doctors and interviewed by the research team. The exclusion criteria were as follows: previous THA, severe acute metabolic, neuromuscular and cardiovascular diseases, excessive obesity (over 45% fat), elevated/high body temperature or other life-threatening situations, infectious diseases, cancer, bleeding, failure of vitally important organs, complete physical exhaustion, mild cognitive impairment or dementia, critical ischemia of the lower limbs, and patients unable to intend the measurement and rehabilitation protocols. All participants were right-handed and had normal or corrected-to-normal vision. All procedures were carried out in accordance with the ethical standards of the 1964 Declaration of Helsinki and were approved by the National Medical Ethics Committee. Written informed consent was obtained from all participants prior to the study, and no payment was provided for participation in this study.

### 2.2. Nonphysical Training Intervention

Participants in the intervention group received next to a standard rehabilitation protocol with physiotherapists an additional nonphysical intervention (AO + MI of locomotor tasks) for approximately 30 minutes per day in the hospital, then 3 times per week in their homes, for a period of 2 months. At the same time and for the same amount of time, participants who were randomly allocated to the placebo control group were asked to actively observe the documentary videos on the television (in hospital and at home). Participants in both groups spent approximately 4–6 days in the hospital before being discharged. All hospital sessions were surveyed and supervised by the authors of the study. Home sessions were supervised by one experimenter during the beginning of the training and then by conference (Skype) video calls. Participants in the control group were also contacted via phone or Skype at least once per week within the period of two months to ensure a comparable level of commitment and to monitor their personal notes regarding the time they spent watching educational documentaries.

Each session of the nonphysical intervention program started with a short relaxation protocol. Afterwards, participants watched video clips presented on a 13-inch tablet PC showing a healthy person filmed from behind performing different locomotor tasks. The difficulty of the presented tasks was progressively increased from week 1 to 8: participants started observing a person walking slowly with assistive devices (e.g., walker and crutches), continuing with normal and fast-paced gait, walking upstairs and downstairs, and walking on narrow (normal and narrow sport bench) and unstable surfaces (soft mats, sand beach, and surfaces covered with snow). When participants reached the level where they could easily imagine themselves performing the presented tasks, an additional task was added such as performing the same locomotor task while holding a glass or a jug full of water. The videos were of different lengths (30 s to 60 s of cyclic locomotor tasks) and were displayed in blocks of 60 to 120 s (see also underneath for more details). Thus, the videos were repeated between two and four times while the participants watched the video and at the same time imagined performing the task that was shown in the video (AO + MI). However, as older adults indicated to have some problems to watch and at the same time “feel the sensations that arise from doing the task” (kinesthetic motor imagery), we presented first the video and asked them to do AO + MI followed by a period in which subjects should close their eyes and imagine the sensations that arise from doing the task (MI). First, this combination of AO + MI and MI was only foreseen in order to familiarize participants with AO + MI. However, this method proved very efficient to maintain the participants' motivation high, and therefore, the entire nonphysical training was conceptualized to maintain blocks of AO + MI followed by the same amount of time for MI. Starting with 60 seconds of AO + MI and 60 seconds of MI, participants in the intervention group were encouraged to prolong their AO + MI and MI sessions up to 120 seconds for each task throughout the whole training period (two months). Altogether 30 different videos were used as shown in Table 2. Thus, during each training day, at least one to two new videos were presented. Before watching the videos, the participants were instructed as follows: “On the following video you will see a locomotor task, which will be repeated several times. Observe this task and imagine performing the task yourself during the entire period of the video. After several repetitions (60–120 s) you will be asked to close your eyes and to continue performing mentally the task until you hear the stop sign. From time to time, I will encourage you performing the task. Now try to relax and when you are ready, press the spacebar to start the video while concentrating as much as possible on the task.”

### 2.3. Outcome Measures

All measurements were carried out in a separate and quiet room to avoid any external disturbances from the hospital environment. All tests were performed twice, prior surgery (PRE measurements) and 60 days postsurgery (POST measurements).

#### 2.3.1. Timed Up and Go Test (TUG)

The TUG test was administered to quantify the functional mobility of patients [29]. Patients were asked to rise from the chair, walk around an obstacle that was 3 m away, and return to take a seat as quickly and safely. A practice trial was given, and then two timed trials were recorded and averaged [30]. Time to completion was monitored with a stopwatch.

#### 2.3.2. Four Step Square Test (FSST)

Patients were asked to perform stepping over 25 mm high obstacles in four different directions as quickly and safely as they could (FSST). A practice trial was given, and two timed trials were performed where the best of both was used for further analysis [31]. Time to completion was monitored with a stopwatch.

#### 2.3.3. Single- and Dual-Task Walking

Spatiotemporal gait parameters were measured with the 2D OptoGait system (Microgate, Bolzano, Italy) in the following four 1-minute conditions in a randomized order: walking at their preferred, self-selected speed; brisk walking to the best of their capacity; and both speeds under a dual-task condition. The dual-task conditions were composed of walking and at the same time subtracting by threes from a randomly chosen number between 400 and 500 (serial 3s). Prior to walking, a familiarization trial was given to each participant. Participants were instructed to subtract as many numbers as possible, with their focus prioritized to task correctness prior to the speed of subtraction. Gait speed and swing-time variability were taken into further consideration [4]. For the cognitive task, the amount of subtracted numbers and errors was monitored for each condition.

#### 2.3.4. Single- and Dual-Task Postural Control

The postural task consisted of standing as still as possible in a tandem foot placement position. Participants were asked to focus on a black point placed approximately one meter in front of them at eye level. A force plate (AMTI HE600600-2k, Advanced Mechanical Technology Inc., Watertown, MA, USA) was used to measure displacements of the center of pressure (COP) in both mediolateral and anteroposterior directions. From these values, total sway path, frequency, and amplitude were calculated separately for each direction. This postural task was either performed as a single task or combined with a secondary cognitive task. The cognitive task and its instructions were identical as for walking (see Section 2.3.3).

### 2.4. Statistical Analyses

The data were analyzed with IBM SPSS Statistics 24.0 software for Windows (SPSS Inc., Chicago, Il, USA). The Shapiro-Wilk test was performed to all datasets to test for normality of distribution. Baseline differences between both groups were assessed with independent sample *t*-test. Interactions were tested by a 2-way analysis of variance (ANOVA) where the group (intervention and control groups) was used as the between subject variable and time (PRE and POST measurements) as the within subject variable. In case of significance, post hoc comparisons with Bonferroni corrections were applied. For ordinal parameters and not-normally distributed data, a Friedman's ANOVA was used. Statistical significance was set at the level of *p* < 0.05.

## 3. Results

From all participants that were included and randomized, 10 participants from the intervention group (age: 64.4 ± 4.1 years; height: 171.8 ± 5.1 cm; weight: 86.6 ± 8.7 kg) and 11 participants from the control group (age: 63.1 ± 5.6 years; height: 168.6 ± 13.8 cm; weight: 76.0 ± 15.7 kg) were considered for statistical analyses. Functional outcome data did not violate normality of distribution as assessed by Shapiro-Wilk test (all *p* ≥ 0.106). Furthermore, the independent sample *t*-test showed no significant differences between the intervention and control groups in any parameter of the baseline characteristics (see Table 1).

### 3.1. TUG Test

**Figure 2:**
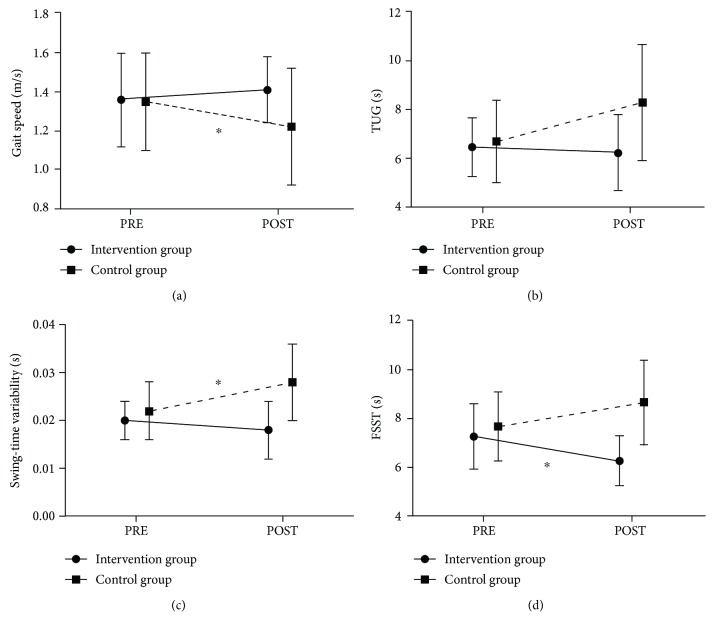
Results from functional locomotor outcome measures (mean ± standard deviation): (a) gait speed in the fast-paced dual-task walking condition; (b) results for the Timed Up and Go test (TUG); (c) swing-time variability in the fast-paced dual-task walking condition; (d) the results for the Four Step Square Test (FSST). Note: ∗ indicates a significant change (*p* < 0.05) in the Bonferroni-corrected post hoc test from PRE to POST.

At PRE, the intervention group did not differ from the control group when performing the TUG (*p* = 0.731). However, there was a significant time × group interaction (*F*_1,19_ = 4.770, *p* = 0.042, *η*^2^ = 0.201). Post hoc analyses revealed that the performance of the intervention group did not differ between PRE and POST (−3.7 ± 14.0%; *p* = 0.427), while there was a trend toward deterioration in the control group, demonstrated as more time spent in TUG at POST (+27.8 ± 37.2%; *p* = 0.061) (see Figure 2). In addition, a significant difference between groups was observed at POST (*p* = 0.031).

### 3.2. FSST Test

At PRE, the intervention group did not differ from the control group when performing the FSST (*p* = 0.498). However, there was a significant time × group interaction (*F*_1,19_ = 11.077, *p* = 0.004, *η*^2^ = 0.368). Post hoc analyses revealed that there was a trend toward an increase in time for completion of FSST in the control group (+14.2 ± 20.9%; *p* = 0.071) while there was a significant reduction in the intervention group at POST (−12.6 ± 12.3%; *p* = 0.014) (see Figure 2). Also, a significant difference between groups was observed at POST (*p* = 0.001).

### 3.3. Single- and Dual-Task Walking

At PRE, no significant differences were found between both groups for any of the assessed gait parameters (all *p* ≥ 0.284). For the self-selected walking speed condition, there were neither significant main (all *p* ≥ 0.429) nor interaction effects (all *p* ≥ 0.150) for gait speed and swing-time variability parameters.

For the single-task fast-paced walking condition, there were no significant interaction effects for gait speed (*p* = 0.132) and swing-time variability (*p* = 0.122). In contrast, during dual-task fast-paced walking, there was a significant time × group interaction for gait speed (*F*_1,19_ = 6.174, *p* = 0.022, *η*^2^ = 0.245). Post hoc tests indicated that the control group significantly decreased gait speed between PRE and POST (−9.26 ± 12.67%; *p* = 0.029) whereas the intervention group nonsignificantly increased their gait speed (+5.15 ± 15.91%; *p* = 0.360) (see Figure 2). Also, for the same parameter at POST, there was a nonsignificant trend between the two groups (*p* = 0.097).

For swing-time variability parameter, there was a significant time × group interaction (*F*_1,19_ = 10.144, *p* = 0.005, *η*^2^ = 0.348). Post hoc tests revealed that the control group significantly increased swing-time variability between PRE and POST (+25.54 ± 23.39%; *p* = 0.006) while the intervention group nonsignificantly reduced swing-time variability (−7.18 ± 30.99%; *p* = 0.315) (see Figure 2). In addition, a significant difference between groups was observed at POST (*p* = 0.004).

For cognitive performance, there were no differences between the two groups at PRE for neither the subtracted numbers (all *p* ≥ 0.482) nor the errors made (all *p* ≥ 0.124). Friedman's ANOVA revealed a significant improvement in subtracted numbers at POST only for the intervention group during both self-selected (*χ*^2^(1) = 8.000, *p* = 0.005) and fast-paced walking (*χ*^2^(1) = 6.000, *p* = 0.014) while no changes were detected for the control group (self-selected: *p* = 0.317; fast-paced: *p* = 0.480). Similarly, for errors made during the serial threes subtraction task, there was a nonsignificant trend for both groups only in the self-selected walking condition: the intervention group nonsignificantly reduced errors (*χ*^2^(1) = 3.000, *p* = 0.083) while there was a trend for an increase in errors for the control group (*χ*^2^(1) = 3.571, *p* = 0.059). Finally, in the fast-paced walking condition, there was neither a change in the number of errors for the control group (*p* = 0.705) nor the intervention group (*p* > 0.999).

### 3.4. Single- and Dual-Task Postural Control

At PRE, no significant differences in any of the postural parameters were found between both groups (all *p* ≥ 0.105).

There were no significant time × group interactions for any of the COP parameters (total sway path, frequency, and amplitude) in both mediolateral and anteroposterior directions, as well as in postural single- and dual-task conditions (all *p* ≥ 0.229).

For the cognitive performance assessed during the serial threes subtraction task, there were no differences between the two groups at PRE, neither for the numbers calculated (*p* = 0.810) nor for the errors made (*p* = 0.819). However, Friedman's ANOVA revealed a significant improvement in subtracted numbers at POST for the intervention group only (*χ*^2^(1) = 9.000, *p* = 0.003) while no changes were observed in the control group (*p* ≥ 0.999). For the errors made during the serial threes subtraction task, there was a nonsignificant trend for reduction of errors in the intervention group (*χ*^2^(1) = 2.778, *p* = 0.096) while there was a nonsignificant trend for an increase in the control group (*χ*^2^(1) = 3.600, *p* = 0.058).

## 4. Discussion

The principal finding of the current study is that two months of additional nonphysical training resulted in better functional and cognitive rehabilitation outcomes in patients with unilateral total hip replacement than with the standard rehabilitation program alone. More specifically, our study supports the feasibility of AO + MI combined with kinesthetic MI in order to accelerate and improve the acute phase of rehabilitation (up to two months after the surgery). The intervention group had better outcomes than the control subjects in physical tests that measured functional mobility and stepping over obstacles, tasks that were actually part of the mental training. In contrast, no differences between the intervention and control groups were found in tasks that were not mentally trained such as static balance. This underlines the task specificity of the mental training intervention.

There is increasing evidence that the combination of AO and MI (AO + MI) leads to greater brain activity in motor areas than either AO or MI alone (for reviews, see [23, 24]). However, the functional implications of these observations for rehabilitation are not clear, yet, as the applicability and effectiveness of AO + MI was scarcely investigated in functional settings. Some of the first studies that concentrated on behavioral outcomes were conducted in the sports domain, where AO + MI was initially entitled “video-guided imagery.” In this context, it was demonstrated that training with AO + MI was more effective in order to learn golf putting [32] or increase elbow flexors strength [33] than the same training with MI. Similarly, Sun et al. [34] demonstrated better rehabilitative outcome in a group practicing concurrent AO + MI than a group that first observed and then imagined the same actions. However, less clear results were obtained after nonphysical balance training with comparable outcomes when using either AO + MI or MI alone [35].

In the present study, we initially wanted to evaluate the influence of AO + MI in a clinical setting by comparing AO + MI with the normal rehabilitation procedure. However, as subjects indicated difficulties to watch and at the same time “feel the sensations that arise from doing the task” (kinesthetic motor imagery), we presented first the video and asked them to do AO + MI followed by a period in which subjects should close their eyes and imagine the sensations that arise from doing the task (MI). Although this kind of mental training intervention intermingles AO + MI and MI, and consequently, the influence of each single mental simulation technique cannot be differentiated, we considered it most important to apply a motivating, comprehensive, and feasible mental training intervention. Furthermore, out of the 405 screened patients, we expected to recruit a higher number of “suitable” participants for the current study that would have allowed the comparison of the current two groups with an additional group performing solely MI. However, although this was not possible, the current approach nevertheless allows the comparison between the standardized (best practice) rehabilitation procedures with the same rehabilitation process amplified with mental simulation of locomotor tasks.

From the behavioral perspective, the current mental simulation training of locomotor tasks in THA patients was highly efficient (a) to counteract surgery-induced impairments that became obvious in the control group (TUG; gait speed and swing-time variability during dual-task walking) and (b) to even improve some motor-cognitive skills already two months after surgery (FSST; cognitive performance during postural tasks and during walking). Another interesting point is the task specificity of the mental simulation approach. Only in tasks that were part of the mental training, participants demonstrated improved task performance compared to the control group whereas in tasks that were not mentally trained, no differences between groups were obvious. Furthermore, the AO + MI training-related effects were mostly seen in the more demanding tasks, such as dual-task walking, where participants needed to perform a secondary cognitive task while walking. The present results are in general agreement with one of our previous studies where nonphysical training led to enhanced performance only in the most attention-demanding walking condition [4].

The underlying mechanisms of mental simulation programs are believed to rely on overlapping brain areas during motor execution and MI as well as during motor execution and AO [14, 22]. In this context, Jeannerod postulated the well-accepted hypothesis that “the motor system is part of a simulation network that is activated under a variety of conditions in relation to action, either self-intended or observed from other individuals” [36]. Recent studies proposed the combination of AO and MI (AO + MI) as this combined approach was found to elicit greater [25, 37] and in some cases even oversummative activity [27, 28] compared to the sum of brain activity during independent AO and independent MI. These findings were interpreted as evidence that neural correlates of MI and AO might merge rather than compete with each other [24]. In this sense, the involvement of distinct brain structures that can solely be activated by either MI or AO [38, 39] would provide supplementary and complementary activation compared to one or the other modality alone. In addition, the activation of common AO and MI brain regions, mostly premotor and motor areas, may induce an overlapping activity [22, 40] that would further augment brain activity with AO + MI. Concerning the execution of AO + MI, it was suggested that the mental simulation of a movement would be facilitated by visual guidance, which allows the participants to update their internal representations. In addition, the visual stimulus generated by AO may help participants to create a visual image, allowing them to focus on kinesthetic modality [41], known to be more effective to activate the motor neural processes than visual imagery only [42]. In line with this, patients in our intervention group reported to be able to perform better kinesthetic MI directly after having seen a video of the task.

With advanced age mental imagery capacities can be altered, especially the temporal features of the imagined action [43]. Our training therefore started with relatively short videos, and the task exposure time was progressively increased. It has been argued that the lack of sensory feedback might be one of the main reasons to explain the decreased ability to perform motor imagery with age [44]. This argues in favor of using AO + MI approaches, since AO may help old adults to compensate for MI ability deficiencies. This is further supported by the finding in elderly subjects that revealed better effectiveness of AO + MI in activating brain areas, than either AO or MI alone [25].

## 5. Conclusion

The present study highlights the benefits of AO + MI interventions for rehabilitation purposes, especially when participants are immobilized after surgery. The results demonstrate that the integrated AO + MI approach was an efficient tool to enhance the functional rehabilitation outcomes of postsurgical orthopedic patients. Remarkably, gains were shown to be exclusively improving the tasks that were actually mentally simulated during the training so that future nonphysical training studies should take into account this task specificity.

In conclusion, AO + MI approaches represent an affordable, safe, and not very time-consuming tool to optimize individual's rehabilitation process. Furthermore, it allows starting the rehabilitation process in the early phase following surgery, when the patient is not able to perform a regular physical training. Once mastered, short sessions of AO + MI performed at home on a regular basis can significantly improve the outcomes of the rehabilitation process, even in frail populations.

## Figures and Tables

**Table 1:** Baseline characteristics of PANGeA hip study participants.

	Intervention group (*N* = 10)	Control group (*N* = 11)	*p* value
Gender	2 women	5 women	
Age (y)	64.4 ± 4.1	63.1 ± 5.6	0.550
Height (cm)	171.8 ± 5.1	168.6 ± 13.8	0.528
Weight (kg)	86.6 ± 8.7	76.0 ± 15.7	0.088
Grip strength, dominant (kg)	35.3 ± 9.1	32.3 ± 15.6	0.605
Total hip replacement, (right side, *N*)	7/10	7/11	
MoCA score	27.9 ± 1.4	28.1 ± 1.4	0.755
Education duration (y)	12.4 ± 3.0	11.8 ± 2.4	0.636

Note: data are mean ± SD. MoCA: Montreal Cognitive Assessment.

**Table 2:** Example of cognitive training intervention blocks for three successive trainings.

Length	AO + MI	Training 1	Training 2	Training 3
2 minutes each	AO + MI	Video 1	Video 2	Video 4
	MI			
	AO + MI			
	MI			
	Break			
	AO + MI	Video 1	Video 3	Video 4
	MI			
	AO + MI			
	MI			
	Break			
	AO + MI	Video 2	Video 3	Video 5
	MI			
	AO + MI			
	MI			

Note: each training session duration was approximately 30 minutes.
